# Activity of contezolid against methicillin-resistant *Staphylococcus aureus* in a rat model of foreign body osteomyelitis

**DOI:** 10.1128/spectrum.02372-24

**Published:** 2025-04-25

**Authors:** Sebastian C. Herren, Melissa J. Karau, Christina Koscianski, Kerryl E. Greenwood-Quaintance, Jayawant N. Mandrekar, Wen Wang, Robin Patel

**Affiliations:** 1 Division of Clinical Microbiology, Department of Laboratory Medicine and Pathology, Mayo Clinic 6915, Rochester, Minnesota, USA; 2 Department of Health Sciences Research, Division of Biomedical Statistics and Informatics, Mayo Clinic 6915, Rochester, Minnesota, USA; 3 Department of Biology, MicuRx Pharmaceuticals Inc., Shanghai, China; 4 Division of Public Health, Infectious Diseases, and Occupational Medicine, Department of Medicine, Mayo Clinic 6915, Rochester, Minnesota, USA; Instituto de Investigacion Sanitaria Gregorio Maranon, Madrid, Spain

**Keywords:** Methicillin-resistant *Staphylococcus aureus *(MRSA), osteomyelitis, contezolid, vancomycin, rifampin, rat

## Abstract

Contezolid has activity against methicillin-resistant *Staphylococcus aureus* (MRSA), a common cause of orthopedic infection. In a rat model of foreign body osteomyelitis, 1.3 log_10_ cfu/g bone and 0.5 log_10_ cfu/k-wire reduction in MRSA was found in tibiae (*P* = 0.0186) and K-wires, respectively, of contezolid-treated compared to untreated rats. No MRSA was recovered from animals receiving rifampin alone or in combination with contezolid or vancomycin. There was no emergence of resistance to contezolid, vancomycin, or rifampin.

## LETTER

Osteomyelitis can occur in conjunction with the presence of a foreign body (1), often as a result of staphylococcal infection (1). The ability of staphylococci to adhere to tissue, bone, and implanted materials as biofilms, persist in host cells, and invade the lacuno-canalicular network provides strategies to avoid the host immune response and antibiotic exposure (1–3). Acquired antimicrobial resistance further complicates therapeutic intervention. Implant-associated osteomyelitis requires surgical debridement, removal of necrotic tissue, and long-term antimicrobial therapy (4, 5).

An inherent risk of prolonged antibiotic treatment is adverse effects, for example, with antibiotics such as linezolid (6). Although active against gram-positive bacteria *in vitro*, myelosuppression and monoamine oxidase inhibition may compromise linezolid’s suitability for long-term antibiotic therapy (6, 7). Contezolid is a new orally administered oxazolidinone active against gram-positive bacteria, *in vitro* and *in vivo* (6, 7), and has a potentially more favorable side effect profile than linezolid (7, 8). Here, contezolid was tested in a rat model of foreign body-associated methicillin-resistant *Staphylococcus aureus* (MRSA) osteomyelitis.

**Fig 1:**
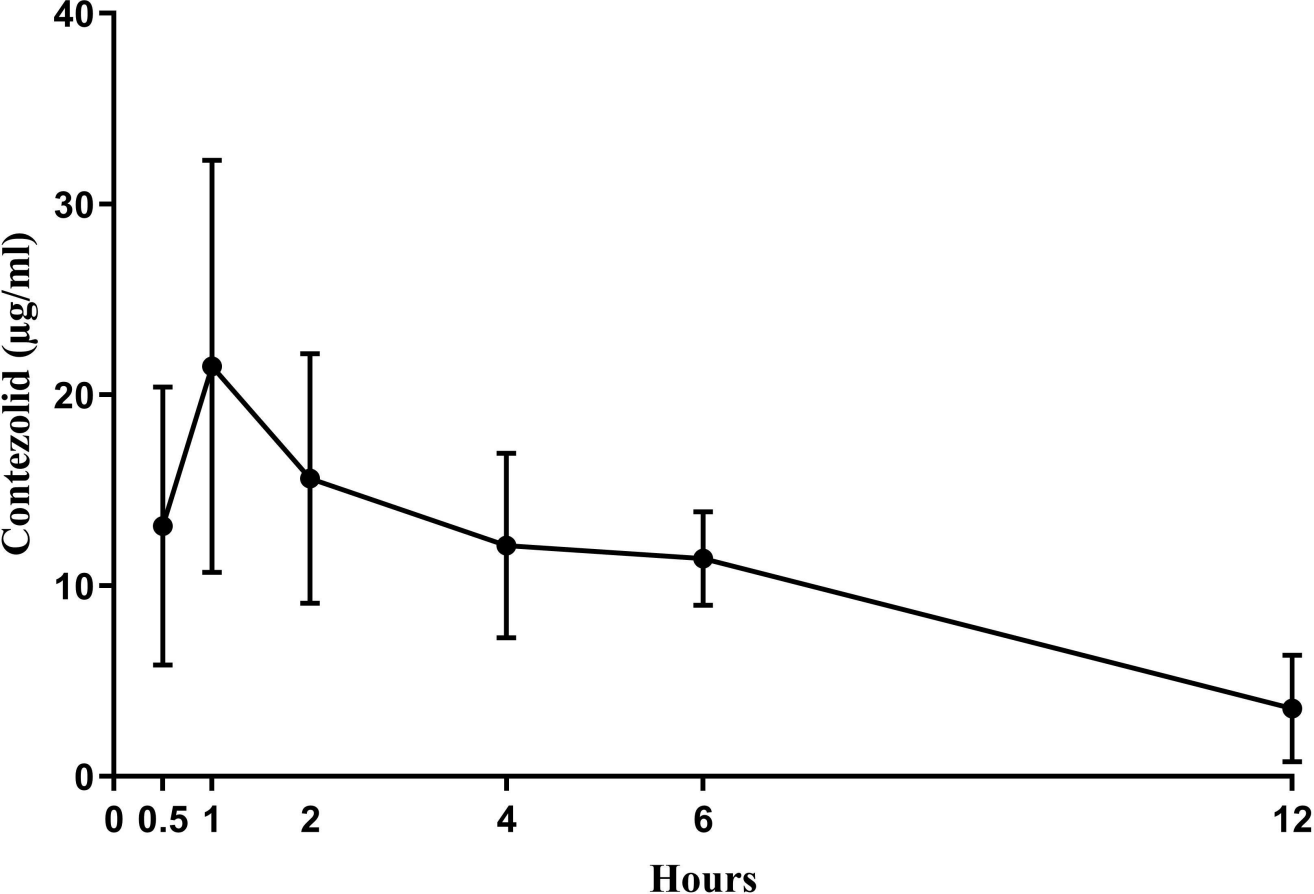
Serum levels after three doses of contezolid (50 mg/kg) orally. Blood was collected from five rats at 0.5, 1, 2, 4, 6, and 12 hours after the third dose and serum concentrations measured by ultrahigh-performance liquid chromatography with tandem mass spectrometry detection (Charles River, Ashland, OH).

To determine serum concentrations in rats, contezolid (50 mg/kg) was administered orally every 12 hours for three doses. Blood was collected at six time points after the third dose. Serum concentrations were measured by ultrahigh-performance liquid chromatography with tandem mass spectrometry detection (Charles River, Ashland, OH) (Fig. 1). The mean (*n* = 5) C_max_, T_max_, and AUC_0-12h_ of contezolid were 21.5 µg/mL, 2 hours, and 123.5 µg*h/mL, respectively, all within the reported range achieved with standard human dosing (6).

MRSA IDRL-6169, recovered from a patient with a hip periprosthetic joint infection, was studied. The minimum inhibitory concentration (MIC), minimum biofilm inhibitory concentration (MBIC), and minimum biofilm bactericidal concentration (MBBC) values of contezolid and vancomycin were 1, 1, and >128 µg/ml, respectively. The MIC, MBIC, and MBBC values of rifampin were ≤0.008, ≤0.008, and 2, respectively.

Experimental chronic foreign body osteomyelitis was established in 60 male Wistar rats using a modification of Zak’s model of experimental osteomyelitis (9, 10). Briefly, the proximal third of the left tibia was surgically exposed, and a 1.5 mm hole was drilled in the medullary cavity. To infect the bone, 10 µL of arachidonic acid (50 µg/mL) and 50 µL of a suspension of 10^6^ cfu of MRSA IDRL-6169 was injected into the bone. Subsequently, a 5 × 1 mm threaded stainless steel Kirschner wire (K-wire) (Zimmer, Warsaw, IN) was implanted in the bone. The hole was covered with dental gypsum and the wound closed.

Treatment was initiated 4 weeks after establishing infection. Animals were randomly assigned to 1 of 5 study arms (12 animals per group): no treatment, contezolid (oral gavage, 50 mg/kg), rifampin (intraperitoneal, 10 mg/kg), contezolid plus rifampin, or vancomycin (intraperitoneal, 100 mg/kg) plus rifampin, with treatment administered every 12 hours over 21 days. Twelve hours after completion of therapy, rats were euthanized, and left tibiae were removed and frozen at −80°C. Tibiae were cryopulverized, and K-wires and bone were separated, with each placed in 1 mL of saline, followed by vortexing and sonication. The resultant sonicate fluid was serially diluted and cultured on sheep blood agar; 0.1 mL was plated on Mueller-Hinton agar containing 4 µg/mL of rifampin, contezolid, or vancomycin to screen for the emergence of antibiotic resistance. Plates were incubated for 24 hours at 37°C. For the cultures with no plate growth, tryptic soy broth was added to the sonicate fluid and incubated for 24 hours at 37°C. If the broth culture detected MRSA, results were recorded as 0.5 log_10_ cfu/g bone or 0.5 log_10_ cfu/K-wire. If the broth culture was negative, values of ≤0.1 log_10_ cfu/g bone or 0.5 log_10_ cfu/K-wire were assigned. Descriptive summaries for each experimental test group were reported as median (minimum, maximum) log_10_ cfu/g bone or 0.5 log_10_ cfu/K-wire.

*A priori* defined comparisons of interest between the five groups were performed using the Wilcoxon rank sum test. Adjustment for multiple comparisons was performed using a false discovery rate approach, which is more powerful than methods like the Bonferroni correction (11). Analysis was performed separately for bone and K-wire measurements; *P* values <0.05 were considered statistically significant. All tests were two-sided. Analysis was performed using SAS software (version 9; SAS Institute, Inc.).

**Fig 2:**
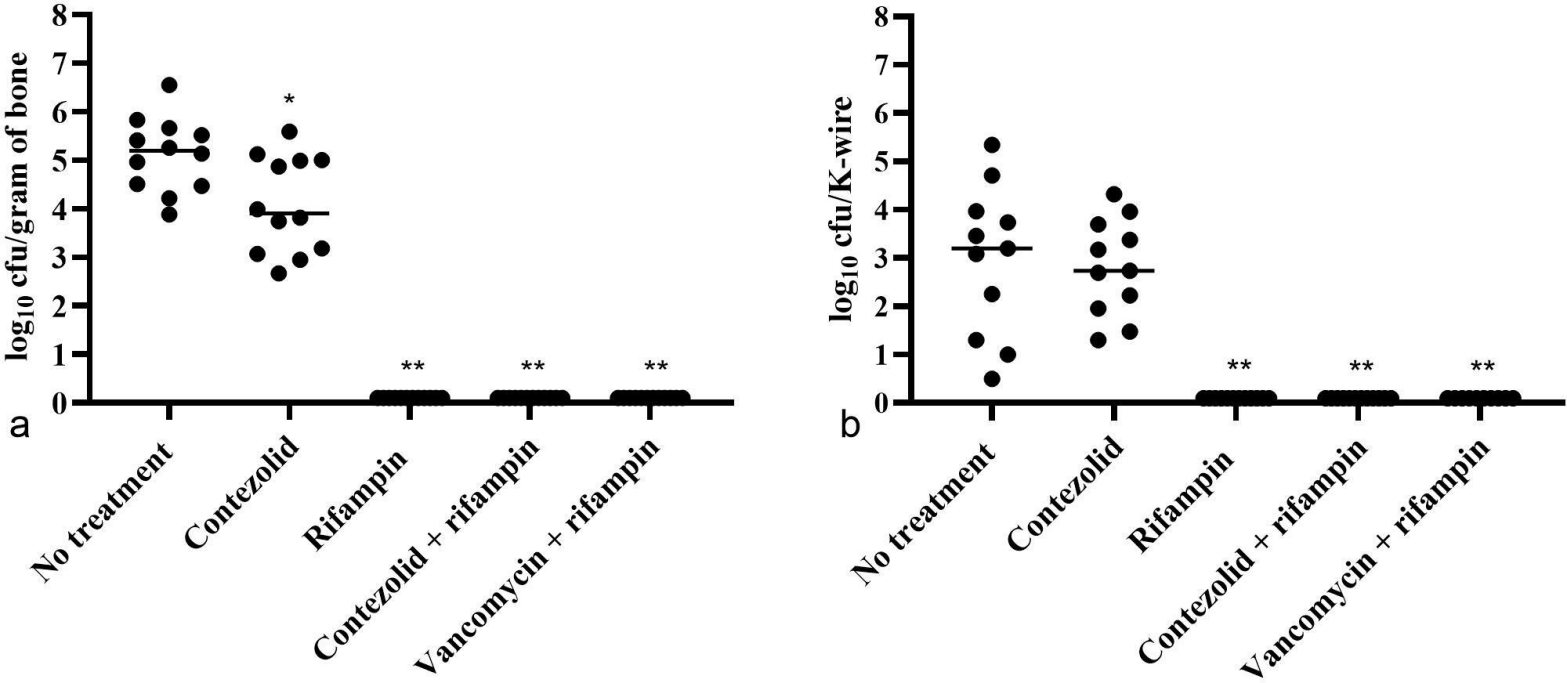
Quantities of MRSA recovered from left tibiae (a) and K-wires (b) after 21 days of treatment. Dots represent values from individual animals. Bars represent median values. *P* values are marked with * for *P* <0.05 and ** for *P* <0.005 when compared to no treatment.

Results of the quantitative cultures of tibiae and K-wires after treatment are shown in Fig. 2. MRSA was recovered from tibiae and K-wires of all untreated animals, with a median (range) of 5.2 (3.9–6.6) log_10_ cfu/g bone and 3.2 (0.5–5.3) log_10_ cfu/K-wire, respectively. There was a 1.3 log_10_ cfu/g bone mean reduction in MRSA recovered from the tibiae of contezolid-treated rats compared to untreated rats (*P* = 0.0186) and a 0.5 log_10_ cfu/K-wire reduction on K-wires (*P* = 0.9564). No MRSA was recovered from the tibiae or K-wires of animals receiving rifampin, alone or in combination with contezolid or vancomycin.

These findings are in line with two previous studies of another oxazolidinone, tedizolid. These investigations studied MRSA and methicillin-resistant *Staphylococcus epidermidis* rat foreign body osteomyelitis; cfu reductions were found in tibiae when tedizolid was administered alone or in combination with rifampin (9, 12). Here, it was shown that exposure to contezolid with and without rifampin yielded significant cfu reductions in tibiae; however, unlike tedizolid, there was no reduction on K-wires in standalone therapy. We previously reported that linezolid in combination with rifampin reduces MRSA cfu quantities in a similar foreign body osteomyelitis model; however, linezolid was not tested alone (13). In a chronic non-foreign body rat osteomyelitis model, linezolid monotherapy was assessed with no difference compared to the tibiae of untreated animals (14).

There was no emergence of resistance to any antibiotic studied here. This is in contrast to previous work, where the emergence of rifampin resistance was found, even in combination with another antibiotic (13). It is unclear whether the different rifampin dosing regimens used may explain these findings.

This preliminary evaluation of contezolid showed a small reduction in MRSA with contezolid monotherapy; this could be due to several factors, including limited antibiotic penetration into bone, biofilm formation on K-wires, and canalicular and intracellular survival. This scenario is not necessarily unique to contezolid (15), emphasizing a need for more effective antimicrobial therapies. Follow-up studies could include pharmacokinetic profiling of contezolid in bone, assessment of surgical debridement and/or removal of the infected implant prior to treatment, and assessment for regrowth of bacteria over time after treatment cessation.

In summary, contezolid reduced MRSA load in bone in a rat foreign body osteomyelitis model, with foreign body retention and no surgical debridement.

## Data Availability

The raw data supporting the conclusions of this article will be made available by the authors on request.
